# Mapping stakeholders’ positioning in the access and delivery partnership value chain: the case of the RTS,S malaria vaccine introduction in Ghana

**DOI:** 10.3389/ijph.2026.1609524

**Published:** 2026-07-24

**Authors:** Xavier Bosch-Capblanch, Kaspar Wyss, Brian Asare, Christian Auer, Evelyn Ansah, Olumide Ogundahunsi, Tuoyo Okorosobo, Margaret Gyapong

**Affiliations:** 1 Swiss Tropical and Public Health Institute (Swiss TPH), Allschwil, Switzerland; 2 University of Basel, Basel, Switzerland; 3 Ghana Ministry of Health, Accra, Ghana; 4 University of Health and Allied Sciences, Ho, Ghana; 5 Consultant, Lagos, Nigeria; 6 Consultant, Abuja, Nigeria

**Keywords:** access and delivery partnership, health policy, malaria vaccine, stakeholders’ analyses, vaccine introduction

## Abstract

**Objectives:**

As population needs continuously evolve new technologies have to find ways to address healthcare challenges. Inputs of key stakeholders are critical when adopting new technologies. Our objective was to map stakeholders along the Access and Delivery Partnership value chain framework (ADP) in relation to the RTS,S malaria vaccine introduction in Ghana.

**Methods:**

Stakeholders engaged with the SAVING consortium were identified and an online questionnaire distributed to map stakeholders positioning in relation to the ADP framework. A workshop was also held to validate findings and to examine the decision processes in adopting new interventions.

**Results:**

31 stakeholders responded the questionnaire reporting their involvement in the ADP framework, most of them identified with stages close to “service delivery”, as opposed to the preceding research and regulatory stages. A few stakeholders were seen as having the highest interest and influence on vaccine introduction.

**Conclusion:**

There is an imbalance between Ghana’s high demand for vaccines and its Research and Development (R&D) capacity, similar to other African countries. Enhancing upstream R&D capacity and fostering more inclusive stakeholder contributions could strengthen the pathway to new technologies adoption.

## Introduction

The World Health Organization (WHO) defines health innovation as the development or refinement of policies, systems, technologies, and delivery methods that enhance health and wellbeing [1]. Embedding innovations such as a new malaria vaccine into existing routine healthcare is inherently complex, context-specific and presents several potential operational pitfalls, such as inadequate integration with existing health infrastructure, supply chains and workflows, [2] inadequate capacity and poor training of healthcare workers. Supply chain weaknesses can compromise vaccine availability and efficacy. Additionally, poor community engagement may lead to mistrust, low acceptance, and vaccine hesitancy. Misalignment between innovation timelines and national health planning cycles can hinder institutional adoption and sustainability. Finally, limited coordination between stakeholders - including government agencies, donors, and implementing partners - can result in fragmented efforts and resource inefficiencies.

Addressing these pitfalls requires realistic planning, inclusive stakeholder engagement and adaptive implementation strategies. Typically, the introduction of health innovations follows a phased approach, which not only supports the creation of solutions but also addresses critical factors necessary for sustainable implementation and scale-up. The example of malaria vaccine illustrates the substantial delays and pitfalls in the process of translating evidence into policy and practices [3]. The Ghana malaria vaccine introduction plan was developed in 2018 and the pilot started in May 2019 [4].

The planning processes around a malaria vaccine embedment in routine health services must actively engage a diverse range of stakeholders - those who influence, are affected by, or have a vested interest in the innovation. Incorporating their insights and experiences ensures that innovations are contextually relevant, ethically grounded, and more likely to be adopted in practice. Stakeholder analysis is thus a critical tool [5, 6].

Firstly, stakeholder analysis helps map the landscape of interests and power dynamics, identifying key actors, such as government agencies, healthcare providers, community leaders, civil society organizations, the private sector, donors and the public. Understanding their roles, motivations, interconnections, power dynamics and concerns enables tailored engagement strategies that foster support and mitigate resistance to the introduction of health innovations. For instance, engaging frontline health workers early in the process can enhance ownership and trust in the vaccine rollout, while involving community leaders and local influencers can improve public acceptance and uptake [7].

Secondly, stakeholder analysis facilitates inclusive planning by integrating diverse perspectives into the design and implementation of innovations. This enhances contextual relevance, ethical acceptability, and alignment with existing workflows and health priorities. In Ghana, such an approach can ensure that the vaccine introduction aligns with national health goals, respects cultural norms, and addresses logistical realities at different levels of the health system.

Finally, stakeholder analysis contributes to sustainability by identifying potential champions and barriers for long-term integration. It supports the development of adaptive strategies that can respond to emerging concerns and evolving stakeholder dynamics.

The SAVING (Sustainable Access and Delivery of New Vaccines in Ghana) consortium [8], funded by the European and Developing Countries Clinical Trials Partnership (EDCTP) and led by the University of Health and Allied Sciences (UHAS), Ghana [9], was formed at the end of 2020 with the aim “to identify and address implementation challenges for the efficient and effective delivery and uptake of new medical interventions”. The focus of the intervention is on implementation research capacity strengthening, enhancing the use of evidence to inform decision-making on the adoption of new health technologies.

**FIGURE 1:**
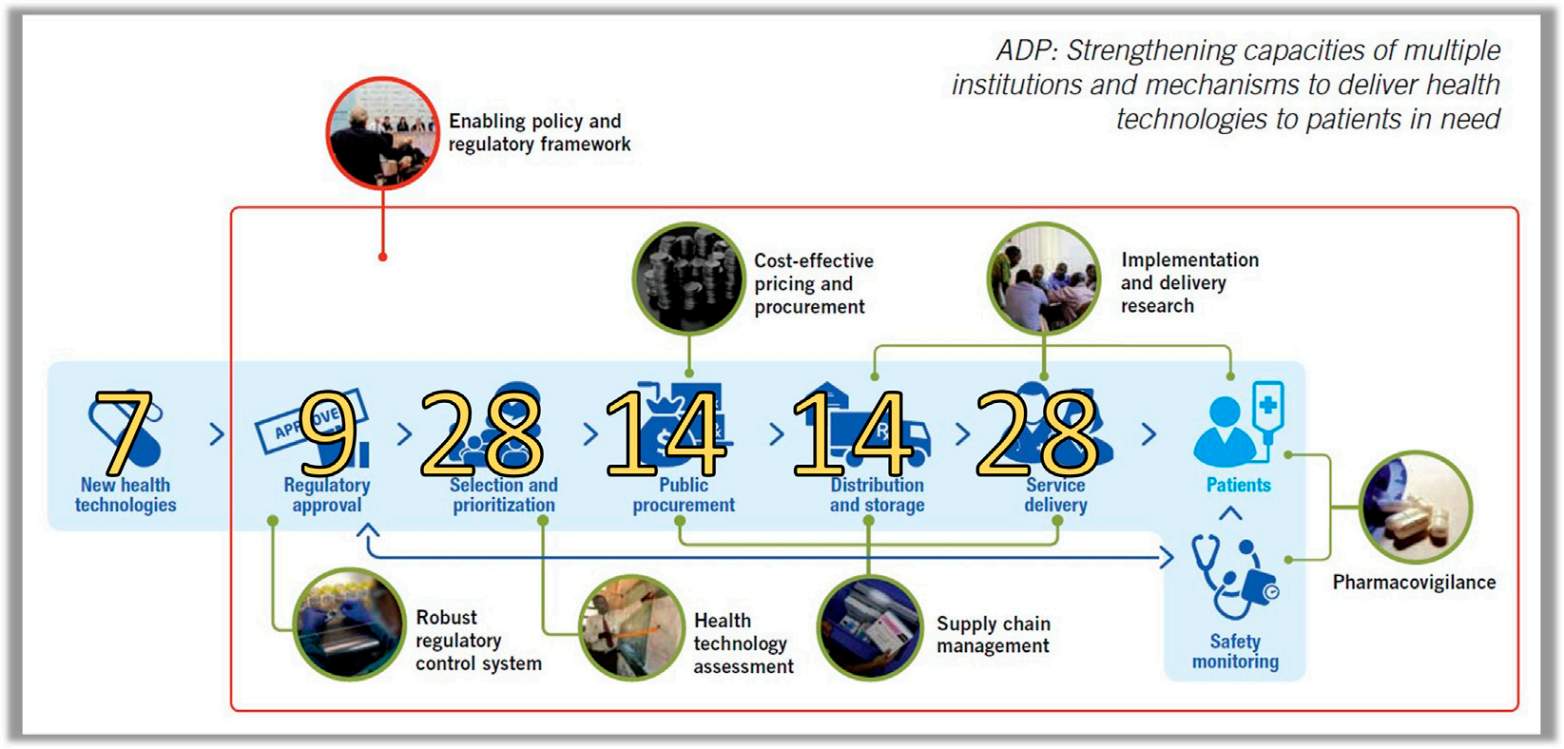
The Access and Deliver Partnership value chain framework (ADP) – self-reported assignment of stakeholders to ADP components (%) (Sustainable Access and Delivery of New Vaccines in Ghana, Ghana, 2020 to 2025). Note: the yellow numbers indicate the proportion of respondents self-reporting their positioning within the seven ADP framework components. Adapted from https://adphealth.org/strengthening-national-health-systems/ (accessed 12/07/2026).

The SAVING setup is inspired by the Access and Delivery Partnership (ADP), a collaborative initiative of the United Nations Development Programme (UNDP) launched in 2013. Its goal is to bridge the gap between innovation and public health outcomes [10]. The ADP framework value chain components include 7 aspects: (i) new medicines, vaccines, diagnostics, (ii) regulatory authorisation, (iii) selection, prioritisation and resource allocation, (iv) public procurement, (v) storage and distribution, (vi) health service delivery and (vii) patients (Figure 1). The ADP supports countries in integrating innovations into national health systems efficiently and equitably and points at a wide range of stakeholders that can support each of the seven components.

In this context, the objective of our research was to map stakeholders against the ADP components and to describe their positioning in relation to the introduction of the RTS,S malaria vaccine in Ghana. By identifying the key stakeholders involved in the vaccine’s introduction and understanding their interrelationships, our study sought to illuminate the dynamics that can either facilitate or impede the adoption of such health innovations. This insight was essential for informing policy recommendations and optimizing the design of implementation strategies in similar contexts.

## Methods

We compiled a list of stakeholders using inputs from SAVING’s consortium members, as well as from their institutional networks and personal contacts of the research team. This included contacts in the Ministry of Health of Ghana, the Ghana Health Service, the Ghana Food and Drugs Authority and the University of Health and Allied Sciences in Ho. Consortium institutions had already carried out a stakeholder mapping and produced their own stakeholder lists, which we used and widened further with our own contacts. We discussed the completeness of the list with consortium leaders and distributed the online questionnaire link by email, following which two reminders were sent to all recipients.

Our questionnaire covered the following domains: informed consent, respondents’ characteristics, self-positioning in relation to the ADP framework components, knowledge on the SAVING Consortium research programme, decision-making processes and positioning in relation to the introduction of the RTS,S malaria vaccine (i.e., influence, interest, implementation capacity, knowledge and funding). The respondents were asked to self-report their perceptions about their own positioning within the seven ADP components. Each component was unfolded into the roles that stakeholders could play in each of them: funding, giving advice, ideation, planning, implementing, analysing or reporting. Multiple answers were allowed.

Additionally, regardless of the positioning of stakeholders in the ADP framework, we asked them in the online questionnaire about their perceptions on which stakeholders or institutions had higher interest, influence (or power), knowledge, implementation involvement, or were able to pay for innovations or new interventions implemented in the Ghanaian health system. This approach was based on the process described in Varvasovszky and Brugha 2000 [6].

Six months after the launch of the online questionnaire, in June 2023, we organised a workshop in Ghana among the SAVING Consortium partner institutions, gathering between 25 and 30 people, to refine and reflect on the stakeholders’ positioning in relation to the ADP framework and the underlying processes in decision-making for the malaria vaccine introduction. We brought the online questionnaire findings to the workshop for their review and validation. The workshop consisted of discussions and group work (i) matching each participant, assuming the role of the stakeholder group he or she belonged to, with the most suitable ADP framework component and (ii) identifying the key stakeholders’ involved in the evidence generation and decision-making for introduction of new interventions in Ghana. The decision-making framework was inspired in a model proposed to describe how innovations find their ways through existing organisations [11, 12].

We calculated absolute frequencies by counting the number of responses and then calculated proportions. Variables included: respondents’ demographics and affiliations, ADP components and the stakeholders assigned to the positioning criteria, mentioned above.

All calculations and graphs were carried out in R [13].

## Results

### Stakeholders’ online questionnaire

The stakeholders’ questionnaire was sent to 121 potential concerned organisations/persons identified by the SAVING partners. 31 responded to the invitation to participate through an online questionnaire, corresponding to a response rate of 26%.

The institutional affiliations of respondents were as follows: UHAS (5, 16%), Food and Drugs Authority (FDA) (5, 16%), Ministry of Health (3, 9%), Ghana Health Service (2, 6%), Swiss Tropical and Public Health Institute (2, 6%), University of Ghana (2, 6%), the World Health Organization (2, 6%), and one each (3%) of the following: a consultant, Expanded Programme of Immunisation, Ghana Coalition of NGOs in Health, Health Access Network, Hope for Future Generations, International Maritime Hospital, Mental Health Authority, National TB Control, PATH and UNDP.

Of the total respondents, 14 (52%) were female and 13 (48%) were male; with 4 respondents not reporting their gender. In terms of qualifications, respondents reported the following degrees: MSc (12, 39%), PhD (7, 23%), graduate without MSc or PhD (6, 19%), senior academic position (e.g., professorship) (4, 13%) and pharmacist (2, 6%).

The positions of respondents in their respective institutions were: senior management other than directorate (13, 42%), mid-level technical position (5, 16%), senior technical position (5, 16%), director (3, 10%), not reported (2, 6%), mid-level management (2, 6%) and deputy chief (1, 3%). The mean time of respondents in their positions were 7 years (range: 1–23 years, median 6 years).

### Stakeholders in the ADP framework

One of the key components of the stakeholder analysis was the stakeholders’ self-reporting about their positioning within the ADP framework, and the domains in which their engagement takes place, depicted in Table 1 and in Figure 1.

**TABLE 1:** Frequency of responses by Access and Deliver Partnership (ADP) framework component and domains (Sustainable Access and Delivery of New Vaccines in Ghana, Ghana, 2020 to 2025).

Domains	Stakeholder positioning on ADP framework						Total
​	New medicines	Regulation	Selection and prioritisation	Procurement	Storage and distribution	Health service delivery	​
Funding	0	0	0	1	0	0	1
Advice	0	0	2	0	1	1	4
Ideate	0	0	1	0	0	0	1
Plan/protocol	0	1	2	0	2	3	8
Implement	1	2	2	2	1	1	9
Analyse	0	1	1	1	0	2	5
Report or document	1	0	3	1	2	3	10
Advocate	1	0	1	1	0	2	5
Total	**3**	**4**	**12**	**6**	**6**	**12**	**43**
Proportion (%)	**7%**	**9%**	**28%**	**14%**	**14%**	**28%**	**100%**

The total number of responses is higher than the number of respondents because multiple responses were allowed. In bold: totals and percentages.

With respect to positioning, there was a higher number of respondents self-reporting on the “right side” of the framework, i.e., the delivery of services. For the left side, closer to the development of new interventions, the most frequent response was “selection and prioritisation.” Both of these top positionings are key components in the implementation of innovations, interventions or services. These were followed by supporting components: “procurement” and “storage and distribution”. The development of “new medicines” or products and regulatory activities are the least prominent.

The main domains across ADP components were “report or document” (10 responses) followed by “implementation” (9 responses), which represent the more operational domains across the ADP framework. “Ideation” is much less represented, as this domain is closer to the development of new technologies rather than to implementation. Funding was mentioned only once, in the component ‘Procurement’.

To illustrate the roles of stakeholders in the ADP framework we also enquired about the knowledge of respondents on the events that led to malaria vaccine introduction in Ghana. Knowledge about malaria vaccine was reported by respondents based on their personal involvement in the different stages of the process, of which we provide some illustrative examples below. For example, an expert who collaborated with PATH mentioned her involvement in phase II and III clinical trials and the work carried out by PATH and the Technical Working Group formed by the Ghana Health Service, leading to the development of a technical brief that guided final decision making by the MoH to participate in the pilot that started in 2019. The expert was responsible for coordinating PATH’s technical assistance in the pilot across Ghana, Malawi and Kenya. Another expert reported being the lead investigator that conducted the qualitative evaluation of the introduction of the malaria vaccine. Another expert had been part of its implementation in the context of pharmacovigilance.

Several respondents mentioned the issue of vaccine efficacy, with diverse views ranging from statements of “low efficacy” to statements related to the achievement of good protection. It was also pointed out that new malaria vaccines (such as R21) may relegate the use of RTS,S, currently in use in Ghana.

Another respondent raised issues about safety and feasibility of its use and acceptance in the routine immunization system. Another respondent reported being involved in monitoring the safety of the RTS,S malaria vaccine.

It was also reported that the WHO focal point in Ghana for the pilot introduction and evaluation of RTS,S provided technical support for activities relating to the pilot implementation programme.

Several respondents explained how the vaccine had been rolled out in Ghana, Malawi and Kenya. In Ghana it was reported that there were several rumours surrounding the implementation of the vaccine, without further details. This affected the uptake of the vaccine in the initial stages of the implementation. Another respondent suggested that the malaria vaccine was introduced as an add-on to other malaria prevention interventions.

Another intervention widely mentioned was COVID-19 vaccination in Ghana. Most of the issues reported around COVID-19 vaccination were descriptive, such as targeted populations, ages and storage conditions. One respondent mentioned vaccine hesitancy as a common phenomenon among the population. Other interventions mentioned included: meningitis, rotavirus and nOPV2 vaccines, mosquito nets, the Med Safety App and Carbetocin for the management of post-partum haemorrhage.

### Stakeholders positioning

In the online survey, respondents were also asked to position stakeholders in relation to the acquisition and implementation of new vaccines, according to several criteria. In terms of influence (or power), the stakeholders most frequently mentioned were the Ministry of Health (5, 16%) and WHO (5, 16%), followed by the FDA, the Ghana Health Service and policymakers in general, all mentioned by two respondents (6%). The following were mentioned once each (3%): care givers, community leaders, EPI, Government, health professional networks, the Malaria Control Programme and PATH.

Stakeholders perceived as having the largest interests were the Ministry of Health (6, 19%), the WHO (3, 10%), caregivers, the Ghana Health Service and the Government (all three: 2, 6%). The Community Pharmacists Association, EPI, the Ghanaian population in general, the National Immunization Technical Advisory Group, the Malaria Control Programme, PATH, researchers in general, the general population and hospitals were mentioned once each (3%).

The questionnaire also enquired about perceptions on stakeholders’ knowledge required to decide about the malaria vaccine introduction, headed by the EPI (5, 16%), the Ministry of Health (4, 13%), the WHO (3, 10%) and health staff (3, 10%); followed by, once each (3%): the FDA, the Ghana Health Service, the Government, Health Professional Networks, the Malaria Control Programme, the Malaria Vaccine Implementation Programme (MVIP), National Immunization Technical Advisory Group, PATH, research institutions and vaccine experts.

In terms of perceptions about stakeholders’ implementation capacity to actually implement the introduction of malaria vaccine in Ghana, this was led by the Ghana Health Service (8, 23%), the EPI (5, 16%), the Ministry of Health (3, 10%), the FDA (2, 6%), followed by once each (3%): frontline health workers, Health Professional Associations, healthcare professionals, the media, national agencies, NGO Leaders and other global immunisation partners.

Finally, the questionnaire also enquired about the perceptions about the capacity of stakeholders to pay for new vaccine implementation.

The most frequently mentioned stakeholders were the Ministry of Health (7, 23%), Gavi (4, 13%), the government, mentioned in general (3, 10%), the WHO (3, 10%) and the Ministry of Finance (2, 6%). The following were mentioned once each (3%): development partners, donors, FDA, multilateral donors, philanthropies and the private sector.

**FIGURE 2:**
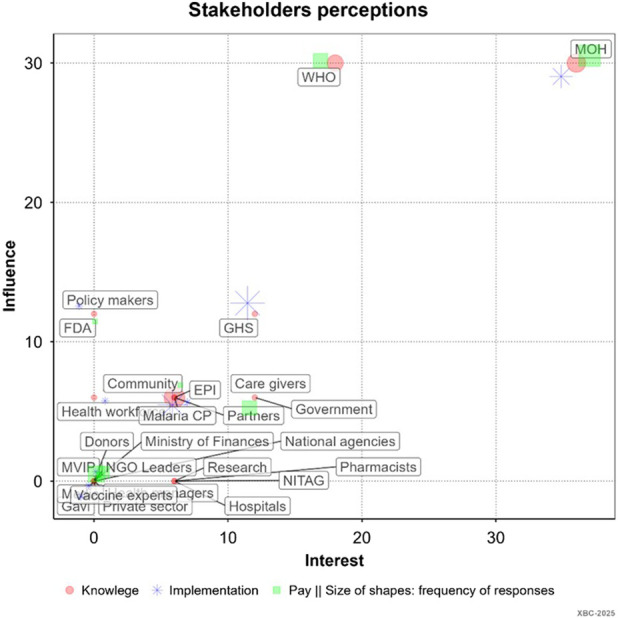
Positioning of stakeholders in relation to malaria introduction influence, interest, knowledge, implementation and capacity to pay (Sustainable Access and Delivery of New Vaccines in Ghana, Ghana, 2020 to 2025).

These positions are graphically represented in Figure 2, where frequencies across different stakeholders and criteria have been added up and shown (i.e., respondents were allowed to mention more than one institution in each positioning criterion). Two institutions, the Ministry of Health and the WHO, stand out. Both are perceived as having similarly high levels of influence (i.e., in their position in relation to the Y axis) and knowledge (i.e., size of circular spot), but the Ministry of Health seems to be perceived as having more interest (i.e., X axis) and capacity to pay (green square). It is important to understand that these figures refer to perceptions and that the positioning is relative to each other (i.e., low interest would only mean that some stakeholders have lower interest than others, but this could be still noticeable interest in absolute terms).

In the quadrant for limited power and moderate interest, we find institutions in the policy making domain: the FDA and Ghana Health Services (GHS). These are seen as having less influence and, strikingly, FDA and policymakers are perceived as having little interest, compared to the GHS. The latter is perceived as having high implementation capacity (i.e., large blue star).

A third group encompassing a substantial number of stakeholders are clustered in the lower-left corner of the graphic, suggesting that they are perceived as having low influence and interest.

Looking at Figure 2, stakeholders could be classified into four different groups (number of stakeholders): (i) “high influence/power, low interest”, (0), “High influence/power, high interest” (2), “Low influence/power, high interest” (0) and “Low influence/power, low interest” (21).

### Role of stakeholders in decision making and implementation

During the SAVING workshop conducted in June 2023, we asked respondents to consider and assign the stakeholders, by order of relevance, to the tasks listed in Table 2. This represents the activities that are related to the production of evidence for decision-making to support each stage in the introduction of new interventions [12].

**TABLE 2:** Assignment of stakeholders in the decision to implementation pathway (Sustainable Access and Delivery of New Vaccines in Ghana, Ghana, 2020 to 2025).

Decisions pathway	Main stakeholders	Stakeholders also mentioned
1. Raise awareness	MOH, WHO	GHS, FDA, programes, NHIA, NGO
2. Issues recommendations	Academia, MOH, GHS, NMIMR	FDA, IRB, PPME, academia, SPH, IHR, NMIMR
3. Advocates for	MHO, programmes, NGO, MOH	GHS, teaching hospitals, programmes, NHIA, FDA, IHR, academia, SPH
4. Approves	FDA, MOH	NHIA, GHS, FDA, parliament
5. Implements	GHS, programmes, NHIA	Programmes, CHAG, teaching hospitals, treatment centres, patients, private health insurance
6. Monitoring and evaluation	GHS, programmes, NHIA	FDA, academia, MOH, GHS, NGOs
7. Further research	GHS-research division, MOH, NHIA, any	Academia, teaching hospitals, programmes, NGOs, IHR, SPH

CHAG: christian health association of ghana; GHS: ghana health services; IHR: institute of health research; MOH: ministry of health; NHIA: national health insurance authority; NGO: Non-Governmental Organisation; NMIMR: noguchi memorial institute for medical research; PPME: policy, Planning, Monitoring and Evaluation Division of GHS; SPH: school of public health.

The results show that MOH and GHS share the main roles in pre-implementation and implementation stages, respectively. Academia is assigned a prominent role in issuing recommendations and carrying out research. Interestingly, the NHIA (National Health Insurance Authority) appears in all stages, except in the issuing of recommendations, while FDA keeps its regulatory role but also plays a role in other domains, except in implementation and research.

## Discussion

We carried out a stakeholder analysis to describe the positioning of several stakeholders in relation to the recent RTS,S malaria vaccine introduction in Ghana, using the ADP framework. This is particularly relevant in the case of the RTS,S malaria vaccine introduction, given the delays in its adoption in policy and practice [3] and the challenges in the availability of malaria vaccines themselves [14].

In Ghana, stakeholders mapped along the ADP framework for the introduction of new vaccines or interventions seem to be concentrated in the regulatory and implementation aspects of it. Interestingly, much lesser presence was assigned to the ‘New Medicines’ component of the ADP framework, even though Ghana was one of the few countries that carried out Randomised Controlled Trials to assess the efficacy of RTS,S [15], contributing to the evidence for its eventual adoption. This reflects, however, the relative imbalance between Africa’s demand for vaccines and its Research and Development (R&D) and production capacity [16] despite the good level of maturity in the regulatory systems of many countries in the African region [17]. Much less presence was found in the components related to the initial phases of innovations, partially attributable to a potential bias in the selection of stakeholders (see ‘limitations’ below). In this context, it is highly relevant that Ghana, in collaboration with Deutsche Gesellschaft für Internationale Zusammenarbeit (GIZ), is taking steps to become a vaccine producer [18].

The positioning of stakeholders, in terms of influence and interest is mainly focused on the MOH, the WHO, and to a lesser extent the GHS, despite that GHS has no normative role. This partially reflects the ‘raison d’être’ of both institutions but may also suggest that there is a concentration of functions in those institutions, when it comes to the introduction of new health interventions or technologies. We could not observe in our analysis the richness of institutions that are portrayed in the institutionalisation of Health Technology Assessment in Ghana, where many stakeholders were identified representing government, academia, multilateral organisations, NGO and beneficiaries [19]. We believe that the workshop in 2023 allowed us to identify a richer scope of stakeholders, since it focused not so much on perceptions about what was going on, but rather on an open discussion about what different stakeholders could contribute to the processes underlying the acquisition of innovations in health. Comparing this with the perceptions in the questionnaire, in real life situations, with limited time and resources, the decision-making cycle is rather concentrated within a few actors over a short period of time, with the potential of missing contributions from other stakeholders. Interestingly, the private sector was largely absent from the workshop’s final conclusions related to decision-making actors.

Finally, while RTS,S introduction has been an excellent platform for Ghana to engage in complex policy and implementation processes [4], it would seem that the COVID-19 pandemic brought more efforts into play: this was reflected in the responses to our questionnaire, as the most frequent mentioned intervention. Indeed, the imbalance in the importance given to COVID-19 vaccine over malaria vaccines is patent at global level [20]: annual deaths attributed to malaria in Ghana is around 20,000 [21] and the total COVID-19 deaths reported from Ghana were 1,463 [22]. All in all, we expect that the COVID-19 events may have brought experience and lessons for malaria vaccine scale-up or the introduction of new malaria vaccines in the near future.

In conclusion, Ghana seems to have the institutional network of stakeholders to support each step in the pathway from new interventions to service delivery. Decision-makers may want to consider (i) how to build capacity at the start of the ADP value chain to ideate, research and produce new interventions and (ii) whether to optimise stakeholders’ involvement in the pathway between evidence to policy (and how, if so).

### Limitations

While the SAVING Consortium provided a unique platform to approach and analyse stakeholders, it also limited the scope of stakeholders to some extent, with a focus on the governmental sector and academia to the detriment of the faith-based and private sector. We cannot rule out selection bias in the scope of the online questionnaire respondents.

The response rate in the online questionnaire (26%), although in the usual range in these types of consultations, was comparatively low and left aside those respondents that had reasons not to respond but who had inputs that could have affected the results of this analysis. However, we counted among the respondents members of the SAVING Consortium, with good knowledge of the health and policy environment in Ghana, and the Ghanaian co-authors could provide a balanced interpretation of the findings. While we may have missed top-level decision-makers, technical level staff have the institutional memory and knowledge to provide better accounts to map stakeholders.

We inquired about the perceptions of respondents, which does not necessarily correspond to an objective assessment of the issues they are presented with. This precludes us from reaching robust conclusions about the actual vaccine introduction processes in Ghana; but allowed us to identify issues that may require further attention and enquiry.

### Conclusions

This stakeholder analysis provided insights into the dynamics shaping the introduction of the RTS,S malaria vaccine in Ghana along the ADP framework. Influence and decision-making appeared concentrated in a few key institutions—MOH, WHO, and GHS—highlighting a possible bottleneck in broader stakeholder engagement. Compared to Health Technology Assessment processes, which involve a wider range of actors, the vaccine introduction pathway appears more centralized. Workshops that focus on ideal scenarios revealed a richer network of stakeholders than real-time decision-making processes suggest. Overall, while Ghana has the institutional architecture to support vaccine introduction, enhancing upstream R&D capacity and fostering more inclusive stakeholder involvement could significantly strengthen the pathway from innovation to implementation.
